# Bilateral Scaphoid Fractures: A Systematic Literature Review

**DOI:** 10.3390/jpm14040424

**Published:** 2024-04-16

**Authors:** Lorenzo D’Itri, Maria Serena Gattuso, Claudio Domenico Cobisi, Massimo Ferruzza, Ludovico Lucenti, Lawrence Camarda

**Affiliations:** Department of Orthopaedics and Traumatology, University of Palermo, 90133 Palermo, Italy; lorenzo.ditri@unipa.it (L.D.); mariaserena.gattuso@you.unipa.it (M.S.G.); claudiodomenico.cobisi@community.unipa.it (C.D.C.); massimoferruzza@libero.it (M.F.); lawrence.camarda@unipa.it (L.C.)

**Keywords:** scaphoid, carpal bones, trauma, surgery, headless compression screws, distal radius fractures

## Abstract

Bilateral scaphoid fractures are rare lesions, warranting a review to synthesize current knowledge, identify gaps, and suggest research directions. Two authors, adhering to PRISMA guidelines, in January 2024 identified 16 case reports (1976–2023). Data extraction included demographics, injury mechanisms, associated injuries, fracture sites, treatments, and outcomes. Among 121 initial outcomes, 16 articles met the criteria, predominantly affecting young people (93.75% males, mean age 22 years). High-energy traumas (75%) often caused associated wrist injuries (68.75%). Most fractures required surgical intervention (68.75%), primarily headless compression screws. Bilateral scaphoid fractures, which are rare but associated with high-energy traumas, commonly involve wrist injuries. Surgical management is often necessary, yielding better outcomes with fewer complications. Further research is essential to understand the epidemiology, optimal management, and long-term results. Early diagnosis and appropriate treatment are crucial for preventing complications and ensuring favorable patient outcomes.

## 1. Introduction

The scaphoid, the largest bone in the proximal carpal row [1], plays a pivotal role in transferring compressive loads from the hand to the forearm and is crucial for maintaining carpal stability [2]. Fractures of the scaphoid are the most frequently encountered fractures among the carpal bones [2], accounting for approximately 2.4% of all wrist fractures in the United States, with an incidence rate of 1.47 per 100,000 person–years [3]. These fractures are more frequent among younger males, particularly athletes and military personnel. They are less common in children, constituting only 3% of all pediatric hand and wrist fractures [4].

Fractures of the scaphoid can be categorized into two primary mechanisms: traumatic and stress-induced. The typical cause is a fall onto an outstretched hand, resulting in forceful hyperextension of the wrist [5,6].

The diagnosis of scaphoid fractures is sometimes challenging. X-rays may miss fractures in up to 25% of cases, leading to delays and unnecessary immobilization. Techniques like CT, MRI, bone scans, and ultrasound offer higher accuracy and quicker results. Ultrasound shows promise due to its availability and accuracy. MRI is considered the best for hidden fractures and associated injuries. MRI or CT scans can be useful for patients with positive clinical findings, alongside repeat X-rays if needed. CT scans and the use of 3D printing can be helpful for diagnosis and especially for surgical treatment planning [7].

Bilateral scaphoid fractures, involving both wrists simultaneously, are exceptionally rare [8] and have been infrequently reported in the literature. The occurrence of bilateral scaphoid fractures can significantly diminish patients’ quality of life, impair functionality, and impose limitations on basic activities of daily living, such as dressing, grooming, and household tasks, but also sports activities and hobbies, thereby highlighting the importance of prompt diagnosis and comprehensive treatment to optimize recovery and minimize disability.

This paper presents a comprehensive review of the literature pertaining to bilateral scaphoid fractures. The objectives of this review were to consolidate the existing knowledge on this uncommon condition, identify literature gaps, and propose directions for future research.

## 2. Materials and Methods

### 2.1. Study Selection

This review of the existing literature adhered to the guidelines outlined in the Preferred Reporting Items for Systematic Reviews and Meta-Analyses (PRISMA) [9].

In January 2024, we conducted searches of PubMed and Web of Science databases using the following research string: “(Bilateral) AND (Scaphoid) AND (Fractures)”. The searches were carried out by two authors (L.D. and M.S.G.).

A standardized data entry form was employed to extract information, including the year of the study, number of patients, sex, age, associated injuries, mechanism of injury, site of fractures, treatment, complications, and outcomes.

Study quality evaluation was performed by two independent reviewers (L.D. and M.S.G.) using the tool provided by Murad et al. [10].

Conflicts about data were resolved by consultation with two senior surgeons (L.C. and L.L.).

### 2.2. Selection Criteria

The selection of studies was based on various inclusion criteria: (1) studies written in the English language; (2) studies of any level of evidence; (3) studies concerning patients with bilateral scaphoid fractures including clinical and radiological diagnosis, type of treatment, outcome, and follow-up.

Several parameters were used as exclusion criteria: (1) inaccessible studies; (2) studies focused on different topics; (3) studies without an accessible abstract; (4) studies with poor scientific methodology. Any duplicates were also excluded.

### 2.3. Data Extraction and Criteria Assessment

**Figure 1:**
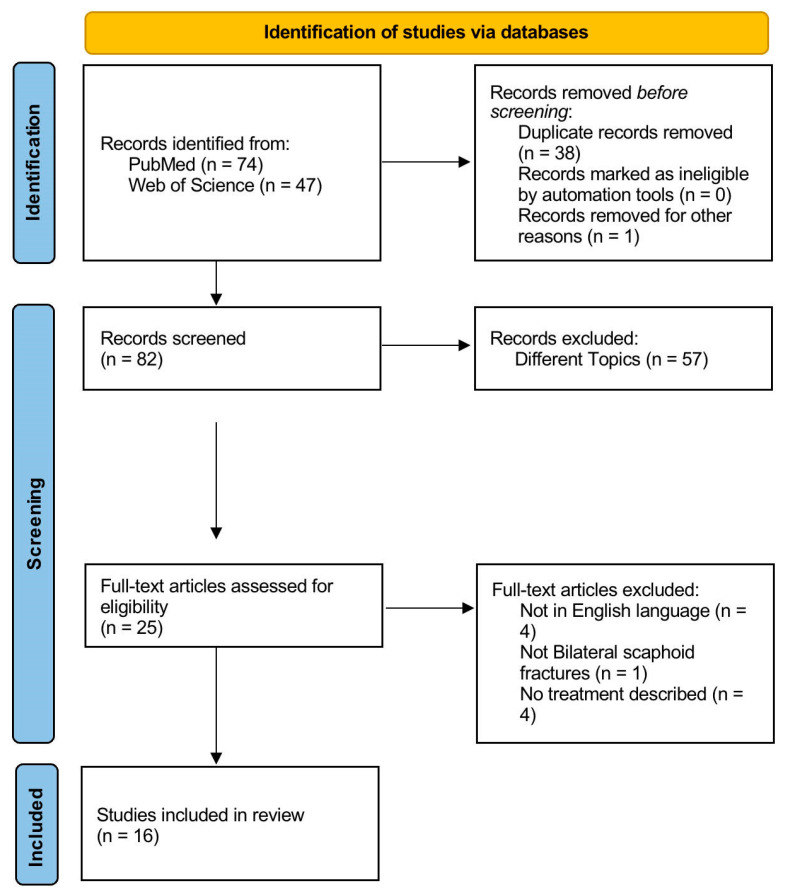
The Preferred Reporting Items for Systematic Reviews and Meta-Analyses (PRISMA) flow chart used for study selection and screening.

Two researchers independently retrieved all information from the text, tables, and figures in the articles. The data were methodically arranged in a specific order: (1) year of publication; (2) first author; (3) paper title; (4) number of patients; (5) sex of patients; (6) age at the injury time; (7) associated lesions; (8) comorbidities; (9) mechanism of injury; (10) CT scan performed; (11) site of fractures; (12) type and timing of treatment; (13) complications; (14) hardware removal; (15) outcomes; (16) follow-up. Conflicts between the two reviewers were resolved by two senior surgeons. The PRISMA flow chart utilized for the selection and screening of studies is shown in Figure 1.

## 3. Results

In the search engine query, a total of 121 outcomes were identified (74 from PubMed and 47 from Web of Science). Subsequently, 38 duplicates and one letter to the author were excluded.

Following the initial screening, a total of 25 articles were selected for a thorough full-text examination. In the final analysis, after an in-depth review of the full texts and scrutiny of the reference lists, a total of 16 articles were chosen following the pre-established criteria.

A flowchart illustrating the selection and screening methodology, following PRISMA guidelines [9], is presented (Figure 1).

Data from the studies were collected. The main parameters are shown in Table 1.

### 3.1. Subjects

All studies, performed between 1976 and 2023, were case reports, each including a single patient with a bilateral scaphoid fracture.

Hence, as shown in Table 1, the cohort comprised 16 participants, including 15 males (93.75%) and 1 female (6.25%). The mean age was 22.13 years.

### 3.2. Mechanism of Injury

In 75% of the cases (n = 12), the injury mechanism involved high-energy trauma. Specifically, 37.5% of the cases (n = 6) resulted from falls from a height [11,12,13,14,20,23], 6.25% (n = 1) were related to a traffic accident [24], 18.75% (n = 3) were associated with falls onto both outstretched hands [15,16,19], and 12.5% (n = 2) were attributed to sports-related trauma (Table 2) [1,21].

Four patients (n = 4; 25%) did not disclose specific traumatic events; instead, they reported multiple minor traumas occurring repeatedly over time, categorizing these fractures as stress fractures [17,18,22,25]. Among these cases, 18.75% (n = 3) could be characterized as sports-related [17,18,22], while, in 5.88% (n = 1), there was no clarification regarding the patient’s engagement in any sports activity [25].

### 3.3. Associated Injuries

In 68.75% of cases (n = 11), associated wrist injuries were identified; of these, 36.36% (n = 4) presented with bilateral distal radius fractures [11,14,21,23], 45.45% (n = 5) had bilateral dislocations of carpal bones [13,16,19,20,23], and 18.18% (n = 2) had bilateral non-scaphoid carpal bone fractures [20,24].

In 6.25% of cases (n = 1), a bilateral proximal radius fracture was described [12], while, in 12.5% of cases (n = 2), injuries to the vertebral column were reported [11,14].

### 3.4. Fracture Site

According to Herbert’s classification for scaphoid fractures, 31.25% of cases (n = 5) exhibited bilateral B2-type fractures [11,14,17,22,25], 29.41% of cases (n = 5) bilateral B4-type fractures [13,16,19,20,23], 6.25% of cases (n = 1) bilateral A2-type fractures [21], and 6.25% (n = 1) bilateral A1-type fractures [12].

Fractures exhibiting discordance bilaterally based on Herbert’s classification were documented in 25% of cases (n = 4). In one case (n = 1; 6.25%), a B2 fracture on the right and a B3 fracture on the left were reported [15]. In another case, an A2 fracture on the right and a B1 fracture on the left were observed [24]. Additionally, two cases presented a B2 fracture on the left and a D2 fracture on the right [1,18].

### 3.5. Treatment

As shown in Table 3, in 31.25% of cases (n = 5), non-surgical management with cast immobilization proved adequate [11,12,16,17,22].

The remaining 68.75% (n = 11) underwent surgery. Among these, 72.73% (n = 8) were treated with headless compression screws [13,14,16,18,19,20,23,24], 9.09% (n = 1) were treated with headless compression screws and bone grafting [1], 9.09% (n = 1) were treated with percutaneous fixation with k-wires [21], and 9.09% (n = 1) underwent a two-stage procedure that included fracture curettage, bone graft from iliac crest, and fixation with headless compression screws, first the right side and then the left side after 6 months [25].

### 3.6. Follow-Up and Outcome

The average follow-up was 15.25 months. Two patients (12.50%) underwent follow-up at 6 months after the trauma; in 50% of cases (n = 8), follow-up was between 6 months and 1 year; in 25% of cases (n = 4), follow-up continued beyond one year.

Complications were absent in 80% of cases (n = 4) treated non-operatively [11,12,15,17]. 

In the remaining 90.91% of surgically managed cases (n = 10), no complications were reported.

## 4. Discussion

Scaphoid fractures represent the most prevalent type of carpal fracture, accounting for 60% of all carpal bone fractures [26]. They mainly occur in young adults and constitute around 3% of all fractures [27]. The scaphoid bone is notably the most frequently fractured carpal bone, with bilateral involvement occurring in about 1% of cases [28,29].

According to a recent review on scaphoid fractures published in 2023 by Almigdad A. et al. [30], it was found the majority of patients affected by bilateral scaphoid fractures were men (15 patients, 93.75%), and the site of the fracture was predominantly in the waist of the scaphoid (84.38%).

In contrast to the findings described by Almigdad et al. [30] for scaphoid fractures, in the present review, it was found that only 43.75% (n = 7) of patients with bilateral scaphoid fractures were between the ages of 25 and 40. Indeed, more than half of the patients with bilateral scaphoid fractures (56.25%, n = 9) fall within the age group under 25 years.

Our analysis revealed a variety of traumatic mechanisms leading to bilateral scaphoid fractures, with falls from height being the most common cause (37.50%). Only 12.50% were due to acute injuries during sports activities. This contrasts with the pattern seen in unilateral scaphoid fractures, where sports-related injuries are more typical [31]. Even when considering stress fractures associated with sports, falls from height remain the predominant cause of bilateral injuries.

As reported for unilateral scaphoid fractures by Wells M.E. et al. [32], the most associated injury with a scaphoid fracture is a distal radius fracture. In our study, we observed that bilateral scaphoid fractures are associated with bilateral distal radius fractures in 25% of cases (n = 4). However, in contrast to the findings for unilateral fractures, the most frequently associated injury, reported in 31.25% of patients (n = 5), is bilateral dislocation of the carpal bones, likely attributable to the increased intensity of trauma, often caused by falls from a height. In two cases described by Afshar A. et al. [20] and Reigstad O. et al. [24], accounting for 12.5% of cases, associated fractures of other carpal bones were identified: in the first case, a bilateral trans-capitate fracture was associated with perilunate dislocation while, in the second case, fractures of the capitate and hamate on the left and only the capitate on the right were observed. Kay R.M. et al. [12] described a single case (6.25%) of a 13-year-old patient with an associated fracture of the proximal radius after a fall from a height of 2 m. Stother I.G. et al. [11] and Ozkan K. et al. [14] delineated the instances of two patients (12.5%) in whom two vertebral injuries were concomitant following a fall from a height. From our results, based on the Herbert classification, the most frequent fracture pattern in bilateral scaphoid fractures was type B2, accounting for 31.25% (n = 5). This finding closely aligns with the study by Duckworth A.D. et al. [33] concerning unilateral scaphoid fractures, where they identified a prevalence of B2 fractures in 36.4% of cases. In 29.41% of cases (n = 5), characterized by the occurrence of bilateral trans-scaphoid perilunate dislocation, a manifestation of type-B4 fractures in accordance with Herbert’s classification was observed in both wrists [13,16,19,20,23].

Meraghni et al. [21] reported a single case (6.25%) involving bilateral scaphoid fractures classified as A2 according to Herbert’s classification, whereas Kay et al. [12] documented a single case (6.25%) featuring bilateral fractures classified as A1. Fractures exhibiting discordance bilaterally based on Herbert’s classification were documented in 25% of cases (n = 4) as follows:

- Yinusa W. et al. [19] described one case (6.25%) with a B2 fracture of the right wrist and a B3 fracture of the left wrist;
- Reigstad et al. [24] described one case (6.25%) with an A2 fracture of the right wrist and a B1 fracture of the left wrist;
- Mohamed Haflah N. H. [18] et al. and Ghargozloo D. [1] et al. each reported two cases (12.5%) of patients presenting with a B2 fracture on the left and a D2 fracture on the right. It was emphasized that the right-sided fracture was asymptomatic in the first case and went undetected for a period of 8 months in the second case.

In our study, we observed that while non-surgical treatment was sufficient for five patients (31.25%), most patients, accounting for 68.75% (n = 11), underwent surgical intervention. All patients treated conservatively did not exhibit dislocations of the carpal bones. In the studies examined, carpal bone dislocation was one of the primary indications for surgical intervention. In the performed surgery, the most frequently employed treatment (72.73%) was fixation using headless compression screws, as also reported in the study by Suh N. et al. [30]. Due to the rotational forces observed at the level of the first row of the carpus, Gray R.R.L et al. [34] considered the possibility of using a double headless compression screw in highly unstable fracture patterns, citing satisfactory results reported in the studies by Garcia et al. [35] and DiPrinzio et al. [36]. In two patients, the use of bone grafts was necessary: in the case described by Ghargozloo et al. [1], this was only performed for the right wrist as the fracture had gone unnoticed, while, in the case reported by Pidemunt G. et al. [25], a bone graft was used bilaterally. The use of autologous bone graft often involves harvesting from a different site, requiring two surgical procedures. In a 2022 study, Vigni G.E. et al. [37] demonstrated improved bone regeneration using an artificial polybutylene succinate scaffold in a rabbit model. This scaffold has already been employed for nerve regeneration in a murine model [38,39]. This application could potentially be considered in the future for cases of bilateral scaphoid fractures in cases not suitable for performing an autologous graft harvesting procedure [40].

As indicated, the mean follow-up duration was 15 months, with 50% of the studies having a follow-up period ranging from 6 months to one year. Follow-up for up to 5 years was described in only one case by Afshar A. et al. [20]. Complications were absent in 80% of cases (n = 4) treated non-operatively [11,12,17,19]. Johnson N.A. et al. [22] reported a singular non-operatively treated case, wherein a subsequent stress fracture of the right scaphoid occurred. This was managed successfully with a single compression headless screw, ultimately achieving resolution. Yildirim C. et al. [16] documented a solitary case within the surgically treated cohort (9.09%) exhibiting a non-painful pseudoarthrosis on the right side. In the remaining 90.91% of surgically managed cases (n = 10), no complications were reported. This highlights that surgical treatment tends to have a lower complication rate compared to conservative treatment, especially because most surgically treated fractures exhibit a pattern of greater instability and/or associated injuries more frequently.

Given the limited sample size, particularly in the conservative treatment group comprising only five patients, it is crucial to approach conclusions with caution. Although the results may appear less favorable, with one complication representing 20% of the patients, extrapolating broader implications remains limited without a larger cohort. While it is plausible that surgical patients may present with more severe injuries, the occurrence of complications should not be presumed solely based on the treatment type.

For non-surgically treated fractures, Almigdad A. et al. [30] indeed recommend a more aggressive management approach and clinical follow-up with a CT scan at 3 months to early detect fractures that are progressing towards nonunion. This allows for the timely referral of these patients to a hand surgery center for optimal case management.

Our study revealed that six patients were under the age of 18 (37.5%); two patients were under 14 years old (12.5%). Pediatric scaphoid fractures are indeed the most common carpal bone fractures, with an annual incidence rate of 11 per 100,000 [41]. On average, they predominantly affect males at around 12.2 years old, while females typically experience them at around 10.3 years old. Scaphoid fractures in boys under 11 years old and girls under 9 years old are indeed rare. Typically, these fractures occur at the distal pole of the scaphoid in children and often heal without complications [41,42,43]. Pediatric scaphoid fractures can sometimes be diagnosed late (in 8% to 29% of cases), leading to uncertainties in treatment approaches. It is not entirely clear when casting is the best option, but it seems preferable over surgery in this group of patients. In our study, one pediatric patient was treated surgically for a bilateral stress fracture of the scaphoid [25].

## 5. Conclusions

Bilateral scaphoid fractures are not very common in the literature and have been documented in only a few patients. This type of injury is often the result of high-energy trauma, typically falls from a height. Given the severity of the trauma, wrist injuries, such as fractures and dislocations, are frequently associated, while elbow injuries are less commonly observed. Patients most prone to experiencing this type of bilateral injury are males aged between 13 and 35 years, with an average age of 22 years. Patients with a history of bilateral wrist injuries should undergo a careful bilateral evaluation to assess the presence of this lesion and treat it appropriately, often through surgical means, to prevent the development of nonunion. It is essential to approach any conclusions with caution due to the small cohort size. Conservative treatment seems to lead to better results than surgery; however, just a few patients treated conservatively were included in this review. Timely and appropriate management strategies are paramount in minimizing the risk of complications and facilitating optimal recovery in cases of bilateral scaphoid fractures. Given the increased complexity and potential for adverse outcomes in bilateral injuries, an adequate approach is imperative to mitigate long-term sequelae. Interdisciplinary collaboration among orthopedic surgeons, radiologists, and rehabilitation specialists is essential to ensure the comprehensive evaluation and management of bilateral wrist injuries. By leveraging collective expertise and resources, healthcare teams can minimize diagnostic oversights and provide timely, effective interventions tailored to each patient’s unique needs. The socioeconomic ramifications of bilateral scaphoid fractures extend beyond individual patient outcomes, encompassing healthcare resource utilization, work-related disability, and financial burdens. Proactive preventive measures and evidence-based treatment strategies are essential not only for optimizing patient care but also for mitigating the broader societal impact of these debilitating injuries.

The literature on this topic is still limited. Our study aims to highlight the possibility that these fractures may happen more frequently due to the increasingly common occurrence of high-energy traumas, emphasizing the importance for medical professionals to systematically assess both wrists for diagnosis and prevent the potential oversight of bilateral injuries. Continued research efforts are essential to expand our understanding of these fractures, including their prevalence, associated risk factors, and optimal treatment approaches. By building upon the existing body of literature, healthcare professionals can enhance diagnostic accuracy and refine treatment protocols to improve patient care.

## Figures and Tables

**Table 1:** Main parameters of each study.

Year	Lead Author	Article	No. of Patients	Sex	Age
1976	Stother I.G. [11]	A report of 3 cases of simultaneous Colles’ and scaphoid fractures	1	Male	26
1999	Kay R.M [12]	Bilateral proximal radial and scaphoid fractures in a child	1	Male	13
2000	Kaneko K. [13]	Bilateral transcapholunate dislocation	1	Male	35
2008	Ozkan K. [14]	Fractures of the bilateral distal radius and scaphoid: a case report	1	Male	28
2010	Yinusa W. [15]	Bilateral Simultaneous Fracture of the Carpal Scaphoid Successfully Treated with Conservative Cast Immobilisation: A Case Report	1	Male	28
2012	Pidemunt G. [12]	Bilateral Stress Fracture of the Carpal Scaphoid: Report in a Child and Review of the Literature	1	Male	13
2014	Yildirim C. [16]	Bilateral dorsal trans-scaphoid perilunate fracture-dislocation: A case report	1	Male	21
2014	Saglam F. [17]	Chronic wrist pain in a goalkeeper; bilateral scaphoid stress fracture: A case report	1	Male	19
2014	Mohamed Haflah N.H. [18]	Bilateral scaphoid stress fracture in a platform diver presenting with unilateral symptoms	1	Male	16
2016	Virani S.R. [19]	A unique case of bilateral trans-scaphoid perilunate dislocation with dislocation of lunate into the forearm	1	Male	35
2019	Afshar A. [20]	Bilateral Scaphocapitate Fracture Syndrome: A Case Report with Long-Term Follow up	1	Male	25
2020	Ghargozloo D. [1]	Traumatic bilateral scaphoid fractures	1	Male	17
2022	Meraghni N. [21]	Bilateral Combined Fractures of the Scaphoid and Distal Radius: A Case Report	1	Female	16
2023	Johnson N.A. [22]	Recurrence of Scaphoid Stress Fracture: A Case Report	1	Male	20
2023	Korkoman A.J [23].	Bilateral greater arc Perilunate injury: A case report	1	Male	25
2023	Reigstad O. [24]	Bilateral carpal pilon-type fractures due to clenched fist trauma: a case report	1	Male	17

**Table 2:** Main characteristics of the trauma and fracture.

Lead Author	Associated Injuries	Mechanism of Fracture	Herbert’s Classification	CT Scan Performed
Stother I.G. [11]	Bilateral distal radius epiphysis fractures; D12 fracture	Traumatic: fall from a height	B2	No
Kay R.M. [12]	Bilateral fractures of the proximal radius	Traumatic: fall from a height	A1	No
Kaneko K. [13]	Bilateral carpal bone dislocations	Traumatic: fall from a height	B4	No
Ozkan K. [14]	Bilateral distal radius epiphysis fractures; L1 fracture	Traumatic: fall from a height	B2	No
Yinusa W. [15]	Bilateral subluxation of the scapho-capito-trapezoid joints	Traumatic: FOOSH *	Right: B2 Left: B3	No
Pidemunt G. [12]	Not described	Stress	B2	Yes
Yildirim C. [16]	Bilateral carpal bone dislocations	Traumatic: FOOSH	B4	No
Saglam F. [17]	Not described	Stress: sports-related	B2	No MRI performed
Mohamed Haflah N.H. [18]	Not described	Stress: sports-related	Right: D2 Left: B2	No
Virani S.R. [19]	Bilateral carpal bone dislocations	Traumatic: FOOSH	B4	Yes
Afshar A. [20]	Bilateral carpal bone dislocations; bilateral trans-capitate fractures	Traumatic: fall from a height	B4	No
Ghargozloo D. [1]	Not described	Traumatic: sports-related	Right: D2 Left: B2	Yes (after 8 months)
Meraghni N. [21]	Bilateral distal radius epiphysis fractures	Traumatic: sports-related	A2	No
Johnson N.A. [22]	Not described	Stress: sports-related	B2	Yes
Korkoman A.J. [23]	Bilateral distal radius epiphysis fractures; bilateral carpal bone dislocations	Traumatic: fall from a height	B4	Yes
Reigstad O. [24]	Capitate and hamate fractures on the left wrist; capitate fracture on the right wrist	Traumatic: road traffic accident	Right: A2 Left: B1	Yes

* FOOSH = fall on outstretched hands.

**Table 3:** Main characteristics of treatments, complications, and outcomes.

Lead Author	Treatment	Complications	Outcome	Follow-Up (in Months)
Stother I.G. [11]	Conservative	Not described	Union	11
Kay R.M. [12]	Conservative	Not described	No pain	1
Kaneko K. [13]	Bilateral headless compression screws	Not described	Union	24
Ozkan K. [14]	Bilateral headless compression screws	Not described	Union	9
Yinusa W. [15]	Conservative	Not described	Union	3
Pidemunt G. [12]	Two-stage (right first, left after 6 months) curettage, iliac crest bone grafting, and fixation with headless compression screws	Not described	No pain	15
Yildirim C. [16]	Bilateral headless compression screws	Non-union of the right scaphoid	Left: union Right: non-union without symptoms	24
Saglam F. [17]	Conservative	Not described	Union	28
Mohamed Haflah N.H. [18]	Bilateral headless compression screws	Not described	Union	4
Virani S.R. [19]	Bilateral headless compression screws	Not described	Union	12
Afshar A. [20]	Bilateral headless compression screws	Not described	Union	60
Ghargozloo D. [1]	Bilateral headless compression screws (right: with non-vascularized bone graft)	Not described	Union	11
Meraghni N. [21]	K-wires percutaneous fixation	Not described	Union	12
Johnson N.A. [22]	Conservative	Recurrent stress fracture of the right scaphoid after 9 months treated with headless compression screws	Union	12
Korkoman A.J. [23]	Bilateral headless compression screws	Not described	Union	12
Reigstad O. [24]	Bilateral headless compression screws	Not described	Union	6

## Data Availability

Not applicable.
